# Report of Committee on Practical Medicine

**Published:** 1858-12

**Authors:** 


(Continued from page 596).
informed us, in a conversation upon this subject, a few days since, that he thinks there is no such disease as milk-sickness, "*sui generis*," but that it is merely a peculiar set of symptoms produced in some seasons more than others, and attending our ordinary bilious fevers, so called.

The Dr. says he has seen very many cases that were called milk-sickness, where all the morbid appearances so commonly described, were seen ; and that after the extreme irritability of the stomach was allayed, the patient would subsequently continue in the condition we find in ordinary miasmatic fevers. From the fact that the usual remedies employed in allaying vomiting and obviating constipation are mentioned as most successful in this disease, there certainly is some reason to conclude that it is merely a peculiar modification of our miasmatic affections, where the cause is so intense as to affect even the brute animals.

We leave the subject with the hope that others in the future may continue to watch its phenomena and discover its true primary cause.

The eighth question in our circular is in reference to cholera infantum. Little or nothing has been received in regard to the disease, and we infer that its prevalence has been limited within the last year.

This disease has prevailed more or less in Joliet for the last four years, and in a form of morbid action that should deeply interest all of us. As its name implies, it is a disease of infancy. It occurs at a time of life when it is extremely difficult to cure any severe form of disease. It occurs, too, during a season of the year when bowel affections are more common in persons of all ages. It is liable to occur among the members of our own households, and of those of our friends most intimate.

We might, with propriety, add to the term cholera infantum that of *Americana*, for it seems to be confined to our country, and principally in cities. For this reason, too, should the American physician feel interested in investigating it. It is not our design to write a dissertation upon this subject, but to present some important considerations, both in diagnosis and treatment, as well as preventive.

It is frequently confounded with ordinary diarrhœa. There are some marks by which it may be distinguished. In the first place, vomiting is almost unfailing at an early stage, and a peculiar sunken appearance, with a deep leaden hue to the face. There is, secondly, great depression of the vital energies very soon after the attack, requiring stimulants and other means to excite action. Thirdly, a peculiar forcible expulsion of the contents of the bowels, as if thrown from a syringe, and a very sickening odor attending the secretions. Other distinguishing features might also be mentioned.

We find cases of cholera infantum most frequent in unhealthy localities, and apartments occupied by too great a number of individuals. The child derives its sustenance from the breast of a mother who also is breathing a vitiated atmosphere, and in most cases has but a limited supply of proper nourishment.

But occasionally we find severe cases of this affection in a family residing in a healthy place, with all necessary appliances of health as to air and food; only the general predisposition exerts an influence in the latter instance.

There is one point which we wish to introduce here in reference to the treatment of this disease which has already been alluded to in our remarks upon dysentery, viz: the avoiding of cathartics and other reducing agents. The judicious use of alteratives, anodynes and tonics, will insure the greatest success in its management. To control the excessive irritability of the stomach, which generally obtains before the physician is called, we have found great benefit from the use of cold to the pit of the stomach and the abdominal region, and allowing the child to take small pieces of ice into the mouth, while warm stimulating applications are made to the extremities. Enemata of cold water alone, or with tr. opii and tannic acid, will frequently control the alvine discharges. As soon as the vomiting is allayed so as to retain medicine upon the stomach, minute doses of some mercurial (if an alterative is indicated), with opii or Dover's powders and sul. quinia, should be administered. The powders are best retained by being placed upon the tongue, to be swallowed with the saliva or washed down by allowing the child to take the breast immediately. The sight of a spoon is to many children most horrid, and it is very desirable not to excite the little patient's opposition if possible. If local inflammation in the stomach can be allayed, the careful use of tonics, with stimulants, such as brandy punch, carb. ammonia, are productive of great benefit. This course must be persevered in with energy, so as to keep up the strength. Most fatal cases result from mere exhaustion of the vital energies.

From the fact that the subjects of the disease in question are most commonly those that are nursing, it would naturally be expected that the condition of the mother greatly modified its character. It is frequently found that a child is laboring under a severe bowel affection while the parent is troubled with constipation. If she takes a cathartic, its influence is sooner manifest upon the infant than herself. Attention directed to her health will soon be favorably appreciated in the object of her care.

If it is true, then, that the effects of a cathartic given to the nurse will be seen in the infant, for the same reason will benefit be derived from the use of tonics and all invigorating agents, either medicinal or dietetic. The state of the mother's mind greatly influences the child, especially in a disease attended with exhaustion. Excessive grief will impair the appetite and prevent sleep. From day to day the child is taking nourishment which has its source by causes above mentioned.

The little sufferer has learned that the breast is the great panacea of all its woes, and frequent appeals in that direction are made. Immediate vomiting may result. An empty stomach still calls for more, and as the secretion is diminished in quantity, and at the same time the strength failing, the exertion necessary to draw the milk tends to increase the exhaustion without much benefit being derived.

The following instance will illustrate the influence of mental causes upon the infant. In the autumn of 1854, a lady called to see an intimate friend who was dying. One of the family had already died of cholera, and new cases were constantly occurring. The mental agitation, together with grief at seeing an intimate friend upon her death-bed, entirely overcame her. She went home and retired. She had a child about a year old, which was at this time perfectly well. He nursed soon after her return, and was taken with severe vomiting and purging in a short time, so as to create alarm.

We were called, and found no evidence of functional derangement. The cause was so apparent that nothing was given, and in a few hours all morbid symptoms subsided. Before night the next day he was as well as ever.

If it is apparent that a child is influenced to any degree by these causes, it must be weaned immediately; and no one can treat diseases of children successfully unless these considerations receive due attention. These hints are thus presented to call the attention of the profession to the observance of such facts.

Too little attention is devoted to the diseases of children. Mothers are apt to think nothing can be done by medicine, and too many medical men are in the habit of saying, "It is but little use to doctor children, and we will leave it to the old women." Such teachings are false, and no one should shun an opportunity to attempt the cure of a child when laboring under disease.

The action of remedies is marked in the case of infants, and the comparative difficulty of diagnosis in their diseases is more than met in their freedom from the whims and prejudices of older persons.

Dr. J. O. Harris, of Ottawa, has kindly furnished the following meteorological record, which accompanies his reply to our circular:

METEOROLOGICAL RECORD FOR 1857, KEPT AT OTTAWA, ILL., BY J. O. HARRIS, M. D. No. 59.

| 1857. | Max'm Temperature | Min'm Temperature. | Maxi'm Mean Temp. | Mini'm Mean Temp. | Monthly Mean Temp. | No. Rain Storms. | Inches Rain. | No. Snow Storms. | Inches Snow. | DISEASES TREATED. |
|---|---|---|---|---|---|---|---|---|---|---|
| Jan.... | 39 | 16 | 32. | 5.33 | 9.82 | 2 | .460 | 3 | 9.50 | Myelitis, Variola, Scarlatina, Colds. |
| Febr'y | 55 | 4 | 48. | 1.33 | 32.65 | 5 | 2.461 | 3 | 6.25 | Phthisis, do. do. Pneumonia, Neuralgia, Remittent Fever. |
| March | 62 | 7 | 47. | 7.66 | 30.30 | 6 | 2.567 | 5 | 6.75 | Delirium Tremens, do. Colds, do. Hepatitis, Myelitis, Remittent and Intermittent Fevers. |
| April.. | 60 | 16 | 52. | 17.66 | 37.09 | 4 | .641 | 7 | 7.75 | Remittent and Intermittent Fevers, Variola, Colds, Pneumonia, Neuralgia. |
| May... | 84 | 32 | 69.66 | 36.33 | 54.55 | 7 | 3.644 | | | Remittent and Intermittent Fevers, Variola, Croup, Neuralgia, Rheumatism, Diarrhœa. |
| June.. | 87 | 44 | 76.66 | 53.33 | 66.66 | 6 | 3.949 | | | Remittent and Intermittent Fever, Typhus Fever, Epilepsy, Hysteria, Rheumatism. |
| July... | 93 | 58 | 85. | 62. | 76.27 | 6 | 3.970 | | | Rem. and In. Fever, Typhus Fever, Diarrhœa. |
| Aug't. | 91 | 52 | 82.86 | 60.66 | 67.95 | 10 | 6.104 | | | Do. do. do. |
| Sept'r. | 84 | 35 | 77.66 | 45.33 | 41.62 | 8 | .891 | | | Do. do. Peritonitis, do. Cancer. |
| Oct.... | 75 | 30 | 64.66 | 32.33 | 49.05 | 7 | 2.684 | | | Do. do. Typhoid Fever, do. Colds. |
| Nov.... | 54 | 4 | 54.66 | 7.33 | 31.86 | 5 | 1.721 | 4 | 7.25 | Do. do. do. Typhoid Pneumonia, Diarrhœa, Variola. |
| Dec.... | 54 | 17 | 46. | 21.33 | 32.90 | 3 | .843 | 4 | 2.75 | Intermittent Fever, Epilepsy, Colds. |
| Totals........ | | | | | | 70 | 29.941 | 26 | 40.25 | |

Annual Mean Temperature, 44.22°.

Dr. Harris writes: "The rate of mortality in typhus was about one in three cases; in what I call typhoid, one in four or five; pneumonia, not more than one in twenty-five. I have scarcely known of a death, within the last six years, from simple remittent or bilious fever, diarrhœa, etc. Scarlatina was quite mild, and I heard of no deaths from that disease. I heard or knew of no *cases* of phthisis."

The Doctor states, in reference to typhoid fever, that he has not seen more than four cases of what he calls genuine typhoid fever in six years. He seems to be more particular in regard to calling our prevailing fevers by that name than many are, and it must be said that his frankness is commendable.

Dr. Harris further states: "It is true that towards the latter part of the season, say from September to January, our (what I call) bilious or remittent fever sometimes assumes a low form, and a few of the symptoms of typhoid fever are seen, such as the dark brown or black tongue, dry teeth and lips, with more or less sordes, and occasionally some stupor, delirium, or tympanites, possibly diarrhœa; but in my own practice, I have seldom, if ever, failed to find, by rising early enough in the morning, a remission more or less perfect. I have failed to discover the rose spots, or any evidence of an affection of Peyer's glands, which Wood declares to be 'as characteristic of this disease as the pustular eruption is of variola;' and I have not failed, as a general thing, to find the fever gone in from five to fourteen days, when every one knows that twenty-one days is a very short *minimum* time for the duration of typhoid fever. And quinine has been my 'sheet anchor.' Day after day, sometimes for a week continuously, I have given from fifteen to twenty grains, in four or five grain doses, usually combined with opium or belladonna in some form; sometimes in solution (I prefer that form if my patients can retain it so), sometimes in pill, and sometimes in powder; but quinine, in some form they must have, and have taken it with a result that has pleased me and my patients,—whether the practice has been scientific or empirical. Of course, I have used other remedies as occasion requires, but I have employed them as the pilot does his helm, while I have been firing up below and getting up steam with quinine."

In reply to the query, What improvements have you to suggest? Dr. Harris remarks:

"None, except that I have heard oil of erigeron, in doses of gtt. v., every two minutes, recommended as certain to arrest promptly *past partum* uterine hæmorrhage." He has had no personal experience as to its virtues, and would like to hear the result where it has been tried.

### STOMATITIS MATERNA.

Respecting this interesting and distressing affection, Dr. D. W. Stormont, of Grand View, Edgar Co., one of the Committee on Practical Medicine, writes:

"During the last four or five years I have occasionally met with stomatitis materna. It has never prevailed extensively or assumed an epidemic form here, nor have the cases generally been severe or unmanageable. The buccal symptoms have usually appeared within a few weeks after delivery; sometimes, however, during the last weeks of gestation. They are uniformly preceded, for an indefinite period, by gastric derangements of various forms and intensity; as acid eructations, a burning in the stomach, spitting up or vomiting a sour fluid or undigested food, a sinking sensation or distension or tenderness at the epigastrium, depraved appetite, costiveness or diarrhœa, etc. After a while pyrosis becomes frequent, the fluid discharged from the stomach is hot or acrid, and there is a scalding sensation in the mouth and throat, which is aggravated by warm drinks. Now apthæ appear on the tongue and inside of the cheeks.

"It is unnecessary to pursue the symptoms farther, or to speculate on the pathology of the disease.

"*Treatment.*—The state of the bowels must be carefully attended to. If there is costiveness, castor oil or seidlitz powders may be administered. After they have been sufficiently acted upon, the pil. rhei. comp. is admirably adapted to keep them regular. If diarrhœa is troublesome, opiates or astringents must be used. A favorite combination with me is,

| | | |
|---|---|---|
| ℞ | Pulv. opii, | grs. vj. |
| | Tannin, | " x. |
| | Pulv. ipecac., | " iv. |

"M. Fiat. Pulveris. xij. One to be taken *pro re nata.*

"It is of primary importance to improve the digestion and increase assimilation. For this purpose a full dose of some preparation of iron may be given three or four times a day. I prefer the following in the order enumerated, viz: muriated tincture, iron by hydrogen, Vallet's mass, prec. carbonate. One of the following pills is ordered *immediately* after each meal:

| | | |
|---|---|---|
| ℞ | Pulv. rhei., | ʒj. |
| | Pulv. ipecac., | ʒj. |
| | Castile soap, | ℈j. |

"Mix with water, and divide into ninety pills, rolling them in calcined magnesia. In passing, I will add that this is a most excellent combination in almost every form of that protean malady—dyspepsia. I am indebted to Dr. Steele for the recipe. The patient is enjoined to use such healthy, nourishing diet as the appetite may crave and the stomach will bear, being guided chiefly by the experience of each case as to the kind and amount to be taken. What disagrees with one seems to be adapted to another. I depend but little on local applications; but a wash for the mouth and throat, of a solution of sulphate of zinc—fifteen or twenty grs. to the ounce of water—is sometimes soothing.

"It is necessary to continue these remedies for several days, or perhaps weeks, after the more obvious symptoms have disappeared, or the disease will return.

"I have seen a development of this disease prevented by a timely relief of the dyspeptic symptoms by the means here detailed. What cases I have seen were in 'miasmatic districts,' and would lead to the inference that the effect of marsh miasm on the general system favors the development of this disease, by the digestive debility and anemia it produces, at a time when inordinate and peculiar demands are made on the blood for the growth of the fœtus or the supply of the lacteal secretion. I have seen nothing 'similar to it in infants or in men.'"

Dr. O. W. Newell, of Plano, Kendall Co., mentions four cases; of one he speaks as follows:

"The first was in July, 1857. Mrs. M. had occasionally an ulcer in the mouth before confinement. It set in immediately after, and I found her, after four weeks, with high fever, no appetite, and no taste in the mouth. The mouth was one white ulcer back to the fauces, and had also extended into the stomach.

"I commenced my treatment by giving iod. potass, in five grain doses, three times a day, and using a saturated solution of the same for a week. Kept the bowels open with cit. magnesia. Saw an amelioration of the symptoms in forty-eight hours, the ulcers assumed a more healthy appearance, the violent pain in throat and stomach ceased. In four days I diminished the dose to grs. ij., and in eight days to gr. j. In sixteen days discharged well. The treatment of the other cases since was similar, and successful in every case.

"I have noticed the disease in infants, in two cases, where they took it from the nurse."

Of the topography of his district, Dr. Newell says, "I am in Kendall Co., between the two streams, Big and Little Rock Creeks. No marshes; no miasmatic influence of a special nature around us. The streams are both swift, and Big Rock now freezes in the winter, and but few streams running into them. As for the cause, it cannot be miasmatic."

Dr. A. J. Crain, of Homer, Champaign Co., also one of the Committee on Practical Medicine, writes the following interesting remarks on stomatitis materna:

"The object of this hasty and imperfect report is to notice a few of the more important characteristics of the above disease, as it prevailed in the practice of the writer during the summer and autumn of 1856, in McLean Co., Illinois; and, although many facts to which I shall refer are directly contrary to the experience of many whose observations have been much more extensive than mine, and whose opinions are entitled to the highest respect, yet they are facts, *and as such*, they are respectfully submitted.

"*First.* The disease prevailed as an epidemic. I do not think there was one lady out of ten who became pregnant during the spring of 1856, but what suffered in some degree during pregnancy or lactation. In most cases, the disease commenced early in pregnancy, and unless arrested, continued with a gradual increase in all the symptoms during gestation and the early months of lactation. The second peculiar characteristic noticed was, that a disease exactly similar in its symptoms and progress occurred in several infants, whose mothers had suffered from the disease during pregnancy.

"In the first case of infantile stomatitis, the infant's mouth presented every symptom of the disease; and what was more remarkable, the anus and lower part of the rectum were excoriated and dotted over with numerous points of ulceration, indicating that the disease had passed through the entire alimentary tract, as in the adult. I examined the infant a few moments after birth, as closely as my powers of observation would admit, and could perceive no difference between the affection as it appeared in the infant and in the mother. This child lived several months. Its mouth got well, but the diarrhœa continued, and it eventually sank under the influence of the disease of the stomach and bowels.

"In the second case, the disease was also intra-uterine in its origin; but the infant had evidently been affected but a short time previous to birth. The stomatitis became more severe in a short time, however, and extended rapidly to the throat, stomach and bowels, producing great difficulty in deglutition, obstinate vomiting and wasting diarrhœa. In a short time deglutition became so difficult, that it was almost impossible to prevail on the little sufferer to swallow even a sup of water. Treatment had but little influence over the disease. The child died when about two weeks old.

"In the third case, the mother had suffered with stomatitis, at intervals, from the third month of utero-gestation. The infant seemed quite well at birth. But three days afterwards it was attacked; and, as in the others, the disease extended rapidly through the whole alimentary tube, and the infant sank gradually under the exhausting influence of the disease.

"The next characteristic noticed during the epidemic was, that the force of the disease was not invariably concentrated upon the mucous membranes, but frequently extended to the deeper-seated tissues. In the first case which proved fatal in my practice, the disease made its appearance during the second month of gestation. I did not see her till the ninth month. At the time of my first visit, I noted the following symptoms:

"A scalding, burning sensation in the mouth, fauces and œsophagus—mastication and deglution extremely painful—a copious discharge of hot, burning saliva. In various parts of the mouth and fauces were to be seen a number of small painful tumors, with hard elevated borders, and surrounded by a circle of inflammation. She had a dull burning pain in the stomach, with frequent vomiting, extreme tenderness in the epigastric region, and a wasting diarrhœa.

"She improved somewhat under treatment, but was attacked on the second day after confinement with acute gastritis, and expired forty-eight hours afterward, perforation of the stomach taking place a few hours after death.

"The next fatal case was that of Mrs. N., pregnant with her second child. She had suffered with stomatitis during her first lactation, and had never enjoyed entire immunity from the disease since it first appeared. The disease had already invaded the stomach and bowels, as was clearly evinced by the symptoms. She gradually improved under treatment, appearing at times almost well; but she suffered frequent relapses till she was confined. A few days after her accouchment she was attacked with all the symptoms of gastro-enteritis, which proved rapidly fatal, perforation of the bowels taking place about twelve hours before death.

"Now, in both of the cases just cited, it is evident that perforation was preceded by ulceration of the mucous and muscular coats of the alimentary tube.

"From the above facts we deduce the following conclusions:

"*First,* That the nomenclature of this disease in not correct, since the disease, in the most marked form, has occurred in infants. Now I am well aware that the children of females who suffer from this disease are generally large, and what might be called well nourished; and I presume that there are many who will be more strongly inclined to doubt the correctness of my diagnosis than to suppose those were well marked cases of infantile stomatitis. In reply to those, I would simply state, that in every case above referred to, the symptoms were quite as well worked as in the adult, and in every essential character the resemblance was complete.

"The second important inference is, that while gestation and lactation, by the drain which they establish, are powerful predisposing causes of stomatitis materna, yet they are not, as has been supposed, the *sine qua non* to its existence; but that any cause which tends to impoverish the blood, and to obstruct the digestive and assimilative processes, may predispose the system to the disease in question.

"*Third,* That while the disease in most cases spends its chief force upon the mucous tissues, still it does not unfrequently implicate the serous and muscular coats of the stomach and bowels, closing the scene by gastric or intestinal perforation. Indeed, there is hardly a tissue or organ which may not become affected during the progress of the disease. Prof. Byford says that he saw a case where permanent deafness of one ear and exfoliation of bone from the mastoid process occurred. Prof. Brainard has seen cases in which there was ulceration of the vagina.

"We would be pleased, did time and space permit, to allude briefly to the pathology of this disease; but as circumstances require brevity, we pass to the consideration of the treatment.

"In this there are two prominent indications—first, to correct the derangement of the digestive organs; second, to restore a healthy quality of blood, and to invigorate the various functions.

"For the fulfilment of the first indication, we are possessed of a variety of means. As we generally find an acid condition of the stomach, and of the salivary and urinary secretions, ant. acids are very necessary, of which the best is b. c. soda or liquor calcis. Alkalies answer the twofold purpose of correcting the acid condition of the stomach, and at the same time neutralizing the acid state of the urine and saliva.

"When there is a strong tendency to the secretion of acid in the stomach, with profuse diarrhœa, the vegetable and mineral astringents will do good service, the best of which are logwood and sub-nitrate of bismuth.

"Dr. Hutchinson, of Ohio, has found the bismuth an excellent remedy, not only in controling the diarrhœa, etc., but also in improving the digestive powers of the stomach; and Dr. W. M. Chambers, of Charleston, relies upon it as the sheet-anchor in the treatment of this disease.

"To meet the second indication, I have found nothing superior to the vegetable and mineral tonics. I have usually prescribed a combination of the extract of gentian, sul. iron and sul. quinine. Professors Evans and Byford, of Chicago, both speak in terms of confidence in relation to the curative properties of c. l. oil in this affection. In regard to local appliances, the influence must of necessity be only temporary; yet I have derived decided temporary relief from the application of the following mixture:

| | | | |
|---|---|---|---|
| ℞ | Tinc. sanguinaria Canadensis, | | |
| | Aqua destillata, | aa, | 1 oz. |
| | Sul. zinci, | | 30 grs. |

"With regard to the diet; in the early stages it should be mild, yet nutritious. But from the first, all acid fruits and vegetables should be avoided. In the advanced stages, when the blood becomes impoverished when it has been drained of its corpuscles, of its albumen, and of the solids generally, and when the system is being rapidly exhausted under the wasting influence of the disease, I have derived great benefit from the more concentrated forms of animal food, and, especially, a diet containing a large amount of albumen, such as eggs, milk, etc. Indeed, I have invariably, through the progress of the disease, recommended an albuminous diet, as I have generally found the blood deficient in albumen from the outset.

"There are many other points connected with this interesting and obscure affection which I would like to notice, but I must postpone their consideration till another opportunity is afforded.

"A. JACKSON CRAIN.

"HOMER, Champaign Co., Ill.,
"April 1st, 1858."

The foregoing articles upon the subject of stomatitis materna, give faithful details of the symptoms ordinarily met with in that affection. Your chairman would beg leave to add some observations in this connection:

First, as to its miasmatic origin. It cannot be said to be strictly of that character, for it is met with in all places, east or west, and in different altitudes and latitudes. It is very evident that the disease is one resulting from a failure in the assimilative process. Occurring chiefly at a time when the mother is called upon to sustain the new being both before and after birth, it is conclusive that it is owing to a want of ability to perform the double responsibility. It is a law of nature in animal life, that all other interests are sacrificed to the good of the one just ready to be brought into the world.

The fœtus in utero must be nourished, even at the expense of her who is called upon to sustain it.

It is well known that all causes that produce exhaustion of the nutritive process affect, in a prominent manner, the mucous membranes. This we see in various other diseases, among which may be named phthisis pulmonalis, where apthæ and colliquative diarrhœa result.

The same is true in the case of the mother after confinement, when the infant is to receive its nourishment from the milk, which is about as direct from the blood as was the nutriment before birth. If the mother secretes healthy milk, a great proportion of the food she digests goes directly to the mammary glands; and if she can furnish enough for herself and her child, without feeling any effects, she of course does not have nursing sore-mouth.

But just in proportion as the child takes her capital stock, so she suffers.

It follows, then, that one of two things must be done in this disease:

1*st*, So act upon the maternal organism by our efforts, as to enable her to sustain both herself and child, or to take the child from the breast and nourish it in some other way.

No mention is made by any of those whose observations are embodied in this report, whether it was necessary to wean the infant. That is the great desideratum; for if we abstract the child from the breast as soon as the mouth becomes sore, recovery will in most cases result without any medication. Your chairman has seen cases frequently, where all the symptoms seen in the mother were witnessed in the child.

These cases appear to occur when the system of the nurse fails to secrete milk of a quality such as will nourish a child. Again, we have seen cases in which the child's mouth became sore from the nipple being in a condition similar to that of the mouth. Great care is requisite that ulcerations of the nipple should be cured on this account, as well as for the comfort of the mother.

Nitrate of silver, internally administered, has, in some cases, been followed with good effects in this disease, to correct the condition of the bowels and stop diarrhœa. As intimated elsewhere in this report, glycerine, with iod. potass and iod. ferri, is confidently recommended, together with brandy or wine, as may best suit, with eggs beat up in it, as a part of the diet. Porter, or lager beer, are excellent, taken as freely as the stomach or brain will admit.

Inasmuch as liquids of all kinds are not well borne, concentrated animal broths, or oyster soup, will serve both as drink and food. A woman suffering from sore-mouth, will, unless strict attention is paid, neglect to take nourishment, both on account of its being painful to eat and want of appetite. For this reason food must be rigidly insisted upon, as well as medicine.

The earlier a woman begins to feel symptoms of this disease, either in gestation or lactation, the more solicitude should be felt in the prompt treatment of the case. We frequently find cases where the mouth will not begin to be sore until the child is some months old. In such cases it is advisable to wean it, unless we are satisfied that remedies are having pretty immediate effects.

It will also be found, in many cases, that the secretion of milk will be morbidly copious, amounting to galactirrhœa; much more milk is furnished than is needful for the infant, and consequently an unnecessary drain is made upon the mother's strength. We have, in such cases, advised the temporary weaning of the child. We say temporary, for it is well known that the secretion from the mammary glands may be restored after being partially or wholly suspended.

A case, now under our treatment, became at one time so severe as to demand the weaning of the child. The milk was very abundant, and the child, although some eight months old, could not take more than half. The mother, notwithstanding the use of tonics and stimulants, continued to lose strength. The infant was taken from the breast, and in less than ten days the milk had nearly ceased. The mother in the meantime had improved so much (and the child showed signs of suffering from the change); that it was again allowed to resume its former nutriment. In order to lessen, if possible, the lacteal secretion at the time of weaning, the following was applied to the breast once or twice daily:

| | | |
|---|---|---|
| ℞ | Atropia, | gr. j. |
| | Aqua distill., | ℥iij. |
| | Acetic acid, | min. ij. |

After the child commenced taking the breast the milk was not so abundant as before, and at present (May 20th), the mother is improving, but takes, daily, beer, and uses the lotion of atropia.

Dr. Hiram Nance, of Lafayette, writes: "In answer to question No. 1, I would say, as I have formerly answered, that sub-remittents and remittents prevail most in summer and autumn, and diseases of the respiratory organs during the winter and spring. I consider it unnecessary to give the causes of these diseases, as they are pretty much settled in the minds of all medical men. The symptoms have not varied materially from what are usual in our miasmatic diseases; the type has been mild and easily controled. A mild cathartic of prot. chlo. hydrarg., combined with pulv. rhei., and followed with quinine in the intermission, or, in place of quinine, sul. cinchonia would, in a few days, remove the disease. It was rarely found necessary to repeat the mercurial. The congestive or pernicious type of intermittents has not prevailed in this part of the country for several years. Our miasmatic diseases are growing less, and fevers are assuming more of a continued type, and leaving off the well-marked periodicity which so well characterized them a few years ago. So mild have been our intermittents and remittents, that I have not known a fatal case during the year.

"I think I have seen less pneumonia during the winter and spring than usual. Some cases proved tedious, resulting in *chronic* pneumonia. The cases that took this turn were those of inflammation of the base of the left lung, connected with pleuritis and bronchitis. I have not bled a patient during the winter or spring suffering under diseases of the respiratory organs. Have treated them with tart. antimony, combined with tr. opii, every two hours, and if the tongue was furred with a yellowish coat, gave a mild mercurial alterative once or twice a day. When the pulse became full and quick, respiration short, and dyspnœa very great, I suspended the tart. antimony and tr. opii, and relied pretty much upon Norwood's tr. veratrum viride. I must say that I have more confidence in it, in the treatment of such types of respiratory diseases, than I have in the vaunted remedy, tartar emetic. I visit my patient to-day and find him with a pulse full and numbering from 120 to 130, respiration quick, cough almost constant. I place him on from five to eight drops of tr. veratrum viride every three hours. On the morrow I see him again; how great the change; pulse down to 70 or 80, respiration free, and cough less annoying.

(To be continued.)

[Concluded from page 644.]

surely possesses tonic properties, and upon inflamed surfaces it appears to exert a decidedly sedative influence; and, indeed, I am inclined to attribute the curative effects which sometimes follow its administration in nervous disorders to its permanent sedative properties. It quiets nervous irritability, and thus allows a more healthy nutrition to take place. Opium, under certain circumstances, seems to act in this way. The case of diabetes which Eberle claims to have cured, was treated substantially upon opium, the whole nervous system having been kept tranquilized by this drug until nature, through the reparative process, overcame the organic disease upon which the affection depended. In my case the ipecac., it is believed, proved highly beneficial in restoring cutaneous transpiration.

And here, gentlemen, I will close, before doing which, however, I will state, that since commencing this dissertation, I have received the July No. of the *Medical News and Library*, in which I find an article to the effect that in hooping-cough the urine is always more or less saccharine. This statement is given on the authority of Dr. Gibb, and seems to have been confirmed by other observers. Now what is the condition of the brain in pertussis? It is evidently in a state of preternatural excitation, and there is strong presumption that the "olivary bodies" are involved in irritation, or perhaps even inflammation. Thus, the position that diabetes depends upon a lesion of the brain receives additional support.

---

NOTE.—In the course of the remarks which the reading of the above paper elicited, the President, Dr. Ritter, related the history of a case of diabetes which he had seen several years previously, in which there was a carious condition of the mastoid processes of the temporal bones. Whether the morbid condition of these bones depended upon disease of the base of the brain, or whether disease of the bones preceded the diabetes, he had no means of knowing. But he knew that the man, who was considerably advanced in life, had diabetes, and also suffered from a carious state of the temporal bones.

I would here render my acknowledgments to Dr. R. B. Denny, a young practitioner of unusually fine promise, for valuable assistance he afforded me in the preparation of this paper. W. M.

